# Tethering of cellulose synthase to microtubules dampens mechano-induced cytoskeletal organization in *Arabidopsis* pavement cells

**DOI:** 10.1038/s41477-022-01218-7

**Published:** 2022-08-18

**Authors:** René Schneider, David W. Ehrhardt, Elliot M. Meyerowitz, Arun Sampathkumar

**Affiliations:** 1 Max Planck Institute of Molecular Plant Physiology, Potsdam, Germany; 2 Plant Physiology Department, Institute of Biochemistry and Biology, University of Potsdam, Potsdam, Germany; 3 Department of Plant Biology, Carnegie Institution for Science, Stanford, CA USA; 4 Department of Biology, Stanford University, Stanford, CA USA; 5 Sainsbury Laboratory, University of Cambridge, Cambridge, UK; 6 Howard Hughes Medical Institute and Division of Biology and Biological Engineering, California Institute of Technology, Pasadena, CA USA

**Keywords:** Cell wall, Plant cytoskeleton, Plant morphogenesis

## Abstract

Mechanical forces control development in plants and animals, acting as cues in pattern formation and as the driving force of morphogenesis. In mammalian cells, molecular assemblies residing at the interface of the cell membrane and the extracellular matrix play an important role in perceiving and transmitting external mechanical signals to trigger physiological responses. Similar processes occur in plants, but there is little understanding of the molecular mechanisms and their genetic basis. Here, we show that the number and movement directions of cellulose synthase complexes (CSCs) at the plasma membrane vary during initial stages of development in the cotyledon epidermis of *Arabidopsis*, closely mirroring the microtubule organization. Uncoupling microtubules and CSCs resulted in enhanced microtubule co-alignment as caused by mechanical stimuli driven either by cell shape or by tissue-scale physical perturbations. Furthermore, micromechanical perturbation resulted in depletion of CSCs from the plasma membrane, suggesting a possible link between cellulose synthase removal from the plasma membrane and microtubule response to mechanical stimuli. Taken together, our results suggest that the interaction of cellulose synthase with cortical microtubules forms a physical continuum between the cell wall, plasma membrane and the cytoskeleton that modulates the mechano-response of the cytoskeleton.

## Abstract (web summary)

The behaviour of cellulose synthase and its interaction with microtubules are affected by mechanical stress. The interaction of cellulose synthase with microtubules impedes the response of microtubules to mechanical stress.

## Main

Living organisms are exposed to mechanical stresses that range from the molecular to the organismal scale. The chemical and physical properties of the extracellular matrix together with its molecular structure largely determine how these stresses are resisted and transmitted as signals into the cellular interior^1^. Integrins, for example, a class of transmembrane receptor proteins assisted by integrin linker proteins, act as molecular tethers between extracellular matrix components and the intracellular actin cytoskeleton in mammalian cells, facilitating transmission of mechanical signals^2^. Similar insights into the extracellular matrix of plants—the cell wall—are limited. Our understanding of the transduction of mechanical stresses to cellular signals in plants includes the observation that the microtubule (MT) cytoskeleton tends to align along the direction of maximal mechanical stress in cells and tissues^3,4^. MTs in turn direct the translocation of cellulose synthase complexes (CSCs) as they synthesize and deposit cellulose microfibrils (CMFs), the stiffest component of the cell wall^5^. This coupling of CMF deposition direction to the stress field acts to reinforce plant cells and to resist the mechanical stresses arising from their own internal turgor pressure and from the differential growth of neighbouring cells, forming a mechanical feedback loop implicated in regulating cell and tissue morphology^6,7^. In epidermal pavement cells (PCs) of cotyledons, MTs are highly ordered (co-parallel) in indenting domains (necks) of negative curvature where anisotropic mechanical stress is also increased, while MTs are disorganized or even absent in positively curved protruding regions (lobes)^4^. More recently, we reported that two MT-associated proteins, CLASP and KATANIN, are required to mediate this cell shape- and stress-associated MT organization^8^. At the tissue scale, KATANIN and other MT-associated proteins such as SPIRAL2 and NEK6 have been implicated in mediating MT organization in the context of mechanical stresses^9–11^. However, quantitative assessment of MT density in negatively curved indentation regions revealed only a moderate correlation to predicted stress^8^, suggesting that mechanical stress-based MT response is subject to mechanical noise, which has been suggested recently to allow organisms to adaptively respond to changing mechanical signals^12^. In addition, MTs by themselves have also been hypothesized to act as sensors, or as part of sensing mechanisms, for mechanical stress^13^. More recent finding suggests that the methyl esterification status of pectin in the cell wall also influences microtubule response to mechanical forces^14^. Apart from this, little information is available on other proteins that mediate or modulate transduction of mechanical signals to MTs.

In cells with cell walls, functional cell shape is determined by patterns of cell wall biosynthesis and by control of how these walls yield to high interior pressure generated by turgor^15,16^. Mutation of cell wall biosynthetic genes has shown the importance of several wall components in generating normal cell shape, especially cellulose and pectins^17–20^; however, cell shape defects are surprisingly modest in comparison with loss of MT function in pharmacological and genetic experiments, which results in more severe perturbation of cell shape in all cell types^21–23^, including the complete abolition of the puzzle-shaped morphology of pavement cells^8^. A recent study based on high temporal resolution and finite element modelling also demonstrated the central importance of MTs and CMF deposition during the initial phases of symmetry breaking in PCs^21^. Besides the process of cell shape regulation, the cell wall is also vital for the propagation of mechanical stresses in tissues^24^. Plant cells are glued to each other by a pectin-rich middle lamella, the loss of which results in adhesion defects between adjacent cells. Nonetheless, how the cell walls communicate through the cell membrane to transduce mechanical stresses to the internal components of the cell such as MTs is unknown.

Here, we describe the presence of a biomechanical continuum between the cell wall, the cellulose synthesizing machinery and the MT cytoskeleton, and we document its involvement in the mechanical stress responses of the MT cytoskeleton. In contrast to mechanical linkages in animal cells involving integrins that act to promote the transduction of mechanical signals to cytoskeletal changes^2^, CSC-mediated mechanical linkage in plants appears to dampen the ability of MTs to respond to mechanical stress.

## CSC patterning and dynamics in pavement cells

**Fig. 1:**
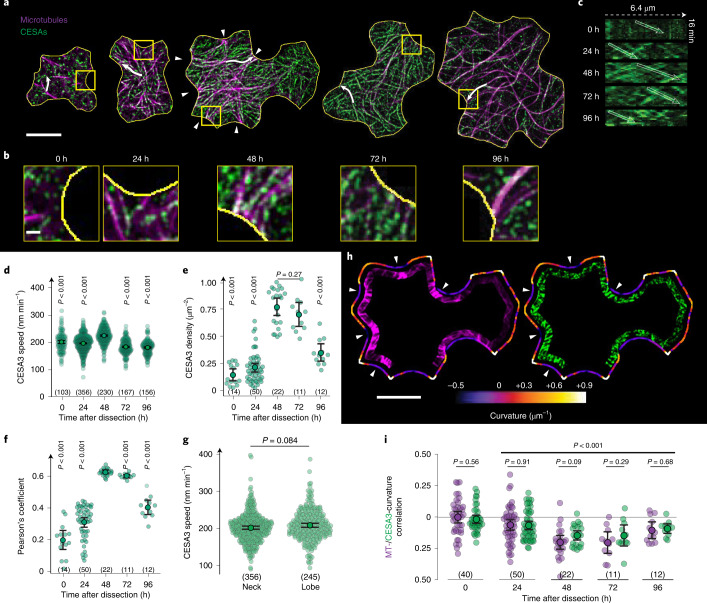
Coordination of MTs and CSCs during pavement cell morphogenesis. **a**, Average projections of MTs (magenta) and CESA3 (green) in single cotyledon cells (yellow outlines) at different time points (hpd). Scale bar, 10 µm. **b**, Zoomed-in images of boxed regions in **a**. Scale bar, 1 µm. **c**, Kymographs along arrows in **a**. **d**–**f**, CESA3 speed (**d**), density (**e**) and Pearson’s co-localization coefficient with MTs (**f**) in cotyledon cells at different time points. In brackets: number of CESAs (**d**) and cells (**e**,**f**). Means ± 95% confidence intervals; Welch’s unpaired *t*-tests (two-tailed, *P* values) relative to 48 hpd. **g**, CESA3 speeds in indentations (necks) and protrusions (lobes), respectively. In brackets: number of CESAs. Means ± 95% confidence intervals; Welch’s unpaired *t*-test (two-tailed, *P* values). **h**, Contour analysis of MTs (magenta) and CESA3s (green) at 48 hpd. Local curvature (coloured bar) depicted as a line plot around the cell outline. Scale bar, 10 µm. **i**, Curvature correlation coefficients of MTs (magenta) and CESA3s (green) for single cotyledon cells at different time points. In brackets: number of cells. Means ± 95% confidence intervals; Welch’s unpaired *t*-test (two-tailed*, P* values) between time points and relative to 0 hpd. Arrowheads depict indentations in which MTs and CESA3s are more abundant as compared with protrusions. See statistics and reproducibility in Methods. Source data

To extract quantitative information regarding the distribution and behaviours of CSCs in relation to the cytoskeleton and cell shape, we imaged CSCs and MTs in cotyledons using GFP-CESA3 and mCHERRY-TUA5 at 24 h stages immediately after dissection from the seed coat (Methods, Supplementary Fig. 1 and Movie 1). Consistent with our previous data, we observed that MTs were relatively sparse at the cell cortex at the beginning of the experiments (0 h, that is, directly after dissection from the seed coat)^8^. Concomitant visualization of CSCs revealed that most did not localize to MTs and migrated independently of MTs (Fig. 1a,f, Supplementary Figs. 2 and 3, and Movie 2). At 48 h post dissection (hpd), a significant transition was observed in which CSC density on the periclinal face of the cell and co-localization with MTs increased threefold (Fig. 1a,b,e,f), while CSC velocities increased modestly but significantly (Fig. 1c,d). Similarly to our previous observations^8^, MT localization to indenting domains was highest (that is, MT signal correlation with positive curvature was more negative) at 48 and 72 hpd, and a similar localization (correlation) was observed for CSCs (Fig. 1h,i). Protruding domains of the cell periphery were not completely devoid of CSCs.

Previously, we hypothesized that the increased presence of MTs in indenting domains at the 48 hpd time point is a consequence of higher local mechanical stresses driven by internal turgor pressure acting on cell shape^8^. Our results here show increased density, velocity and co-localization of CSCs to MTs at similar developmental stages. We hypothesize that an upregulation of cellulose synthesis would allow cells to withstand high-turgor-driven mechanical stress experienced during stages of rapid growth and cell size increase. To test the possibility that CSC migration rates and thus also synthesis rates^25,26^ are elevated in subcellular domains of high mechanical stress, we compared the migration rate of CSCs in indenting versus protruding domains. We found no significant differences in speed, which suggests that the rate of cellulose synthesis is independent of subcellular localization and of the subcellular level or anisotropy of mechanical stress in these cells (Fig. 1g and Supplementary Fig. 4). We can then conclude that during early stages of PC development, a transition occurs as cell shape evolves in which MTs become more localized to negative cell curvature while CSC association with MTs increases as do their abundance and velocity. These changes indicate that cellulose synthesis becomes more concentrated in indenting regions as they form, where mechanical stress is the highest. However, CSC velocity, and thus the activity of individual CSCs, do not appear to correlate with mechanical stress level differences in different subcellular domains.

## MTs regulate cell shape-dependent CSC patterning

**Fig. 2:**
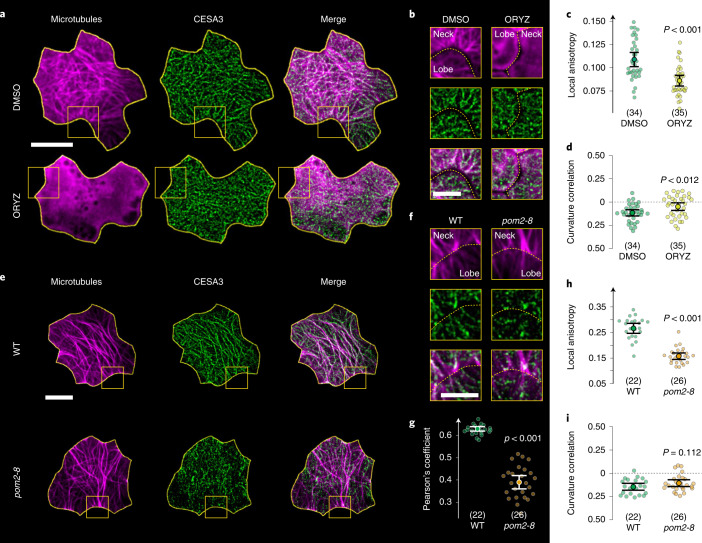
Pharmacological and genetic perturbation of MT guidance of CSCs disrupts their organization at indentations. **a**, Projections of MTs/tubulin (magenta), CESA3 (green) and merged channels in single cotyledon cells (yellow outlines) at 48 hpd after 4 h DMSO (top) and oryzalin (ORYZ) treatment (bottom). Scale bar, 10 µm. **b**, Zoom-ins of regions marked in **a** showing more organized CESA3 trajectories in DMSO- as compared with ORYZ-treated cells. Scale bar, 5 µm. **c**,**d**, Local CESA3 anisotropy (**c**) within a 1.8 µm margin from the cell contour, and CESA3-curvature correlation coefficients (**d**) in DMSO- and ORYZ-treated cells. In brackets: number of cells. Means ± 95% confidence intervals; Welch’s unpaired *t*-test (two-tailed*, P* values). **e**, Projections of MTs (magenta), CESA3 (green) and merged channels in single cotyledon cells (yellow outlines) at 48 hpd for wild-type (top) and *pom2-8* mutant cells (bottom). Scale bar, 10 µm. **f**, Zoom-ins of regions highlighted in **e** showing more organized CESA3 trajectories in wild type as compared with *pom2-8*. Scale bar, 5 µm. **g**, Co-localization of CESA3 and MTs as measured by Pearson’s correlation coefficients in wild type and *pom2-8*. In brackets: number of cells. Means ± 95% confidence intervals; Welch’s unpaired *t*-test (two-tailed*, P* values). **h**,**i**, Local CESA3 anisotropy (**h**) and CESA3-curvature correlation coefficients (**i**) in wild type and *pom2-8*. In brackets: number of cells. Means ± 95% confidence intervals; Welch’s unpaired *t*-test (two-tailed*, P* values). See statistics and reproducibility in Methods. Source data

Given the similarity in the subcellular distribution pattern of MTs and CSCs relative to cell shape, we asked whether cell shape in itself could direct spatial patterning of CSCs independently of MT function. To test this possibility, we first treated PCs with the MT-depolymerizing drug oryzalin. Oryzalin treatment and MT disassembly had no significant effect on CSC density at the cell membrane (1.11 ± 0.17 and 1.04 ± 0.22 for 34 mock and 35 Oryzalin-treated cells, mean ± s.d., *P* > 0.14), but CSCs in the oryzalin-treated cells were significantly less associated with regions of negative curvature as compared with mock-treated cells (Fig. 2a–d and Supplementary Fig. 5a,b). In addition, we observed that the CSC trajectories in indentations appeared to be more disordered in oryzalin-treated cells (Fig. 2a,b), an observation that was supported quantitatively by measurement of CSC track anisotropy along the entire cellular outline (Fig. 2c and Supplementary Movie 3).

As a second means to test the role of MTs in determining CSC distribution with respect to cell curvature, we used the cellulose synthase interacting1 (CSI1/POM2) mutant *pom2-8* that partly decouples CSCs and MTs^27–29^. We found that CSC trajectories *in pom2-8* were more disordered at the PM (Fig. 2e), including along the cell periphery (Fig. 2f,h), as compared with wild type, although the distribution of MTs seemed relatively unperturbed (compare MT images in Fig. 2e,f, Supplementary Fig. 5c,d and Movie 4). Additionally, we found CSCs to have a significantly reduced co-localization with MTs in PCs (Fig. 2g). Despite this reduced co-localization, CSC association with indenting domains was not significantly different from that measured in wild-type cells (Fig. 2i). Taken together, we conclude from these findings that cell shape alone is not sufficient to determine CSC distribution. However, since we did not measure a significant effect on the distribution of CSCs with respect to cell curvature in the *pom2-8* mutant, it is possible that CSI1/POM2-mediated tethering of CSCs to MTs does not explain all MT function in organizing CSC with respect to cell shape.

## CSC-MT association hinders cell shape-dependent MT ordering

**Fig. 3:**
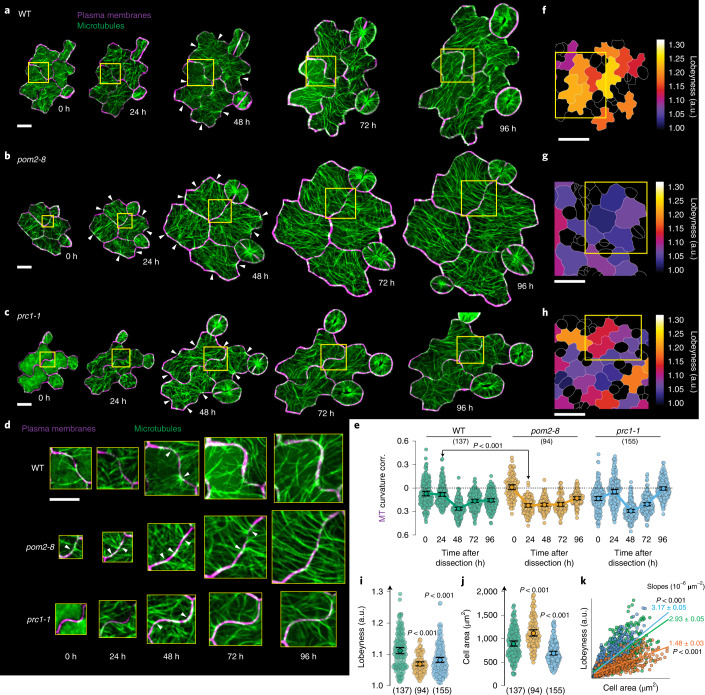
Disruption of CSC tethering to MTs impairs cell geometry-dependent control of MT organization, impacting cell shape complexity. **a**–**c**, Projections of MTs (green) and PMs (single optical section along the anticlinal face in magenta) in a long-term cell tracking experiment for wild type (**a**), *pom2-8* (**b**) and *prc1-1* (**c**). Scale bar, 10 µm. **d**, Zoomed-in images of boxed regions in **a**–**c**. Arrowheads indicate focused MT bundles in necks. Scale bar, 10 µm. **e**, MT-curvature correlation for wild type, *pom2-8* and *prc1-1*. In brackets: number of cells. Means ± 95% confidence intervals; Welch’s unpaired *t*-test (two-tailed*, P* values) between wild type and *pom2-8 at* 24 hpd. **f**–**h** Lobeyness heat maps at 96 hpd for wild type (**f**), *pom2-8* (**g**) and *prc1-1* (**h**). Scale bar, 10 µm. Box indicates region depicted in **a**–**c**. **i**,**j**, Lobeyness (**i**) and cell area (**j**) at 96 hpd. In brackets: number of analysed cells. Means ± 95% confidence intervals; Welch’s unpaired *t*-test (two-tailed*, P* values). **k**, Scatterplot of lobeyness against cell area (from **i** and **j**) for all time points (0 to 96 hpd). Linear regressions reveal the rate of shape formation. Means ± 95% confidence intervals. Welch’s unpaired *t*-test (two-tailed, *P* values) relative to wild type. See statistics and reproducibility in Methods. Source data

We next investigated the possible role of MT-CSC tethering in cell shape-dependent MT organization. To explore this question, we performed a long-term tracking experiment in which we followed individual PCs in wild type and *pom2-8* (Fig. 3a,b and Supplementary Fig. 6). *pom2-8* has been reported to have a lower cellulose content^27,28^, which could increase mechanical stresses in PCs. To help distinguish between effects of disrupted tethering of CSCs and MTs and cellulose content, we also tested the CESA6 mutant *prc1-1* that reduces the amount of cellulose without impairing CSC-MT tethering^30^ (Fig. 3c). Quantitative assessment showed that MTs in the *pom2-8* mutant cells correlated significantly better with indentations already at 24 h as compared with wild type despite reduced cell shape complexity (Fig. 3a–e). This increase in MT-curvature correlation was not observed in *prc1-1* cells at 24 h (Fig. 3e). These findings indicate that MTs are more highly associated with cell shape in the *pom2-8* mutant at this stage than in wild-type cells. This behaviour may be dependent on reduced MT-CSC tethering in the *pom2-8* mutant as opposed to a change in mechanical stress due to lower cellulose content.

Morphologically, we found that *pom2-8* mutant cells were significantly less complex in cell shape at 96 hpd (Fig. 3f–i) and that their area as viewed from above was larger compared with those in wild type and *prc1-1* (Fig. 3j). These results contrast with phenotypic assessments performed on mature pavement cells of the *pom2* mutant^21^. However, it is important to consider the developmental progression of the cells when performing such comparisons. To obtain such a developmental perspective of shape change, we plotted the cell size of all cells tracked from 0 to 96 hpd against their respective cell shape complexity (lobeyness)^31^. We confirmed that *pom2-8* mutant cells produce complex cell shapes at a significantly slower rate in terms of areal growth than wild-type and *prc1-1* mutant cells (Fig. 3k). While the cells of the *prc1-1* mutant appear less complex in shape at 96 hpd, consideration of cell size in addition to cell shape complexity revealed that unlike the *pom2-8* mutants, *prc1-1* is not drastically different from the wild type (Fig. 3k). However, it should be noted that CESA1 mutant *any1* has been previously documented to show simpler cell shapes^17,20,21^. Taken together, these results indicate that disruption of MT-CSC tethering enhances cell shape-dependent organization of MTs, yet the reduced MT-CSC coordination results in formation of simpler cell shapes.

## CSC-MT interaction attenuates mechano-response

**Fig. 4:**
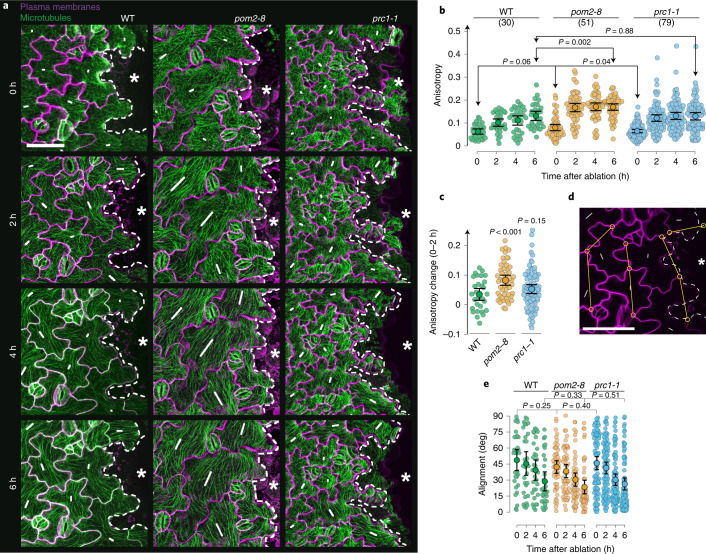
Genetic perturbation of CSC tethering to MTs attenuates mechano-transduction to MTs. **a**, Projections of MTs (green) and PMs (single optical section along the anticlinal face magenta) of cotyledon PCs neighbouring the ablation site (asterisk and dashed line) at 96 hpd in wild type (left), *pom2-8* (middle) and *prc1-1* (right). White lines indicate MT orientation and anisotropy (proportional to length) in each cell. Scale bar, 50 µm. **b**, MT anisotropy over 6 h after ablation for wild type, *pom2-8* and *prc1-1*. In brackets: number of cells. Means ± 95% confidence intervals; Welch’s unpaired *t*-test (two-tailed*, P* values) between start (0 h) and end (6 h), respectively. **c**, Initial anisotropy changes between 0 h and 2 h for the three genotypes. Means ± 95% confidence intervals; Welch’s unpaired *t*-test (two-tailed*, P* values) relative to wild type for the same cells as specified in **b**. **d**, Definition of MT co-alignment: angular difference between the orientation of the MT array (white lines) of each cell (magenta outlines) relative to the nearest polygonal stretch of the ablation site (yellow lines). Scale bar, 50 µm. **e**, MT co-alignment with the ablation site (0 degree is perfect co-alignment) over 6 h after ablation for wild type, *pom2-8* and *prc1-1*. Means ± 95% confidence intervals; Welch’s unpaired *t*-test (two-tailed*, P* values) between 0 h and 6 h, respectively, from the same cells as specified in **b**. See statistics and reproducibility in Methods. Source data

Having established a link between MT-CSC tethering and the ability of cells to organize their MTs relative to cell shape and mechanical stresses driven by cell shape, we next asked whether MT-CSC linkage also affects MT organization in response to mechanical stresses at the tissue scale by performing ablation experiments that alter tissue-scale mechanical stresses without influencing cell shape. In wild type, as reported previously^4^, MTs responded by co-aligning with the border of the ablation site and by a gradual increase in MT ordering as measured by MT anisotropy^32^ (Fig. 4a(left),d). MTs in the *pom2-8* mutant showed an overall stronger MT response in terms of anisotropy (Fig. 4a(middle)), which was predominantly achieved during the first 2 h after ablation (Fig. 4b,c), while the wound-edge alignment dynamics were not significantly different from that of wild type (Fig. 4e). This behaviour was independent of cell wall cellulose content as the *prc1-1* mutant showed a response similar to that of wild type (Fig. 4a(right)–c,e). This is consistent with similar results obtained in the shoot apical meristem of *cev1* (CESA3) mutants, where no difference in MT response to mechanical perturbations was observed in comparison with the wild type^33^. These results indicate that uncoupling of CSCs and MTs causes MTs to show increased response to changes in tissue-scale mechanical stresses.

## Changes to mechanical stress reduce CSC density

**Fig. 5:**
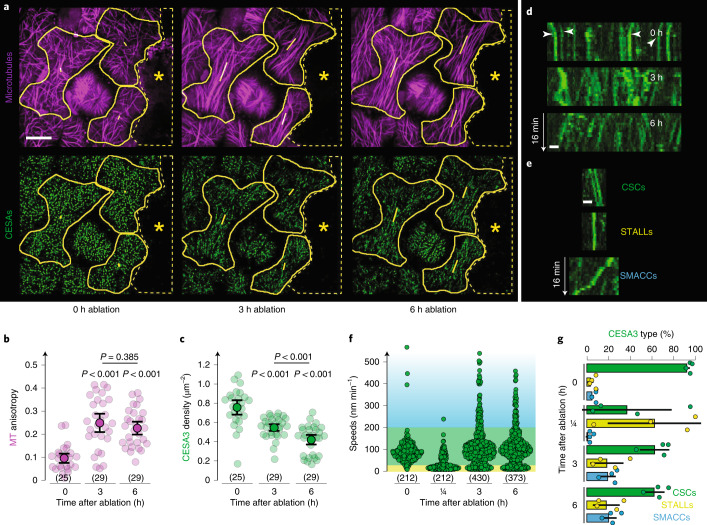
Mechanical perturbation by ablation affects CSC trajectories and tracking behaviour. **a**, Projections of MTs (magenta) and CESA3s (green) in cotyledon PCs at the 48 h time point at 0, 3 and 6 h after ablation (dashed area and asterisk). Approximate cell outlines in yellow. MT orientation and anisotropy indicated by the yellow lines inside each cell. Scale bar, 10 µm. **b**,**c**, MT anisotropy (**b**) and CESA3 density (**c**) in cells neighbouring the ablation site at 0, 3 and 6 h post ablation. In brackets: number of cells. Means ± 95% confidence intervals; Welch’s unpaired *t*-test (two-tailed*, P* values) relative to the 0 h time point. **d**, Kymographs at different timepoints after ablation. Arrowheads depict initially moving CESAs that stall in the 0 h kymograph. Scale bar, 1 µm. **e**, Classification of CESA foci into steadily moving (termed CSCs, green), immobile (termed STALLs, yellow) and erratically moving (termed SMACCs, blue) subpopulations. Scale bar, 1 µm. **f**, Speed distributions of all CESA3 foci directly after ablation (0 h) and after 15 min (=¼ h), 3 h and 6 h. Coloured background depicts the cut-off velocities for CSCs (green, 25–200 nm min^−1^), STALLs (yellow, speed <25 nm min^−1^) and SMACCs (blue, speed >200 nm min^−1^). In brackets: number of CSCs examined in the same cells as in **b** and **c**. **g**, Fraction of CSCs, STALLs and SMACCs over the time course of the ablation experiment. Means ± s.d. *N* = 4 cotyledons with number of cells and CSCs given in **b**, **c** and **f**, respectively. See statistics and reproducibility in Methods. Source data

On the basis of the above results, we hypothesized that a reduction in the presence of CSCs at the PM could facilitate MT response to changes in mechanical stress. To further explore the connection between CSC density and MT rearrangement, we modulated tissue-scale mechanical stresses by means of physical ablation of cells and monitored MT and CSC response over time in adjacent cells. Consistent with previous results, a significant increase in MT anisotropy was observed at 3 h post ablation (Fig. 5a,b). Concomitant observation of GFP-CESA3 showed that while trajectories changed along similar directions as that of MTs, the density of GFP-CESA3 decreased after ablation in cells adjacent to the ablated one (Fig. 5a,c). Closer examination of GFP-CESA3 dynamics revealed that the migrating GFP-CESA3 foci abruptly stopped immediately after ablation (Supplementary Movie 5) but gradually began to move again at later time points. (Fig. 5d and Supplementary Movie 6). Quantitative assessment of dynamics indicated an overall decrease in speeds of GFP-CESA3 foci after mechanical perturbation. In addition, we observed an increased presence of small CSC compartments (SMACCs) that exhibit higher velocities and rapid direction changes (Fig. 5d–g). To rule out irreversible damage from the mechanical wounding as a cause of these observed changes, we performed a control experiment in which we applied and removed a gentle compression to cotyledon cells for 3 h^4^. As expected, we saw that the compression resulted in an increase in MT anisotropy after 3 h of compression (Supplementary Fig. 7a,b) and observed further that GFP-CESA3 densities were also reduced by this treatment (Supplementary Fig. 7c,d). However, these effects were reversed 3 h after release of the compressive forces, with the MTs returning to a more isotropic organization along with increasing densities of CSCs (compare Supplementary Fig. 7d,e). Transient changes to mechanical forces in the *pom2-8* mutants showed that the density of GFP-CESA3 foci also decreased, but significantly more than what was seen in the wild type (Supplementary Fig. 8). This indicates that MT interaction with CSC to a certain extent contributes to the presence of GFP-CESA3 foci after transient changes to mechanical forces. Together with the observation of MT behaviour in the *pom2-8* mutants, these results suggest that changes in mechanical stresses result in decreased CSC density at the PM, which in turn may facilitate MT response to mechanical stress fields.

## Discussion

Using a quantitative live-cell imaging approach, we reveal molecular insights into the dynamic behaviour of CSCs during early stages of PC development. Imaging of PCs within the first hour after dissection from the seed coat revealed the existence of a CSC population that migrate independently of MTs. CSCs displayed increased density and co-localization with MTs and exhibited faster movement at later developmental stages. These observations indicate that CSC presence, velocity and association with MTs are developmentally controlled similar to those reported in etiolated hypocotyl cells^34^.

Previously, we showed that MTs are more abundant in indenting domains—regions that by their shape have been shown to experience elevated anisotropic mechanical stresses^4^. Quantitative assessment of CSC presence along the cell contour indicates that CSCs are also predominantly present in indenting domains as they form. This suggests an upregulation of CSC density, which, together with a greater propensity of MT guidance, would increase the amount (and appropriate direction) of cellulose deposition, effectively countering increased mechanical stress in these regions. The gradual decrease in CSC association with regions of negative curvature also indicates a shift from the initial morphogenetic process of elaboration of local cellular curvature and form towards more isotropic growth reported during older developmental stages^21,35^. Further, transient depolymerization of MTs and molecular uncoupling of CSC and MTs (as in the *pom2*-8 mutant) show that cell shape and associated mechanical stress are in themselves not sufficient to influence the patterning of CSC movements but rather that such patterns rely on the ability of MTs to direct CSCs. These results largely support the view of a mechanical stress-based feedback loop that regulates CMF deposition in an MT-mediated fashion in developing cells^6^. However, assessment of CSC velocities in indenting and protruding domains did not reveal any significant differences in CSC migration rates, suggesting that the rate of cellulose synthesis per CSC is not influenced by mechanical stresses and is under developmental regulation.

Recent meso-scale, coarse-grained molecular dynamics simulations demonstrated that tensional forces are heterogeneously distributed within modelled composite cell walls, with CMFs withstanding most of the generated stresses^5^. The model also shows that tensile forces are transmitted primarily via CMF-CMF interactions. The nascent CMF physically interacts with existing CMFs in the cell wall while still being associated with the CSC residing at the PM. This association acts as a potential medium to transduce tensile forces from the cell wall to proteins and protein complexes such as CSCs. In addition, CSCs are connected to the MT cytoskeleton via CSI1/POM2, a configuration that bears similarity to the molecular interconnection between extracellular matrix, integrins and cytoskeleton described for mammalian systems^2^. While it has been hypothesized that a physical continuum between cell wall, PM proteins and MTs in plants could play a role in the transduction of mechanical stress, details are lacking^13,36^. Our results show that the uncoupling of MTs and CSCs to a certain extent enhances the ability of MTs to orient heterogeneously in response to cell shape, which imposes heterogeneous mechanical stress. Apart from the CMF-CSC/CSI1-MT continuum, interaction between plasma membrane-localized receptor kinase FERONIA and guanosine nucleotide exchange factor GEF14 was shown to activate plant Rho-related GTPases ROP6, potentially influencing MT organization in response to mechanical stress^14^. However, an independent study suggests that MT response to changes in mechanical stress occurs independent of FERONIA action^37^. Therefore, unlike the animal integrin receptors, the role of receptor kinases in mediating MT response to mechanical stress in plants requires further investigation.

The interface between the extracellular matrix and the cell’s internal components is considered as the central hub in transducing mechanical signals from the exterior to intracellular components in Mammalia^1^. Here, we demonstrate a functionally analogous mechanism in plants where changes to mechanical stresses transiently impact the dynamics and abundance of the CSCs that link the external CMFs to the MTs via CSI1/POM2. Mechanical stresses are also shown to stabilize such junctions and are hypothesized to modulate the structural properties of proteins by altering their binding affinities, thereby eliciting a biochemical response^1^. Abrupt changes to mechanical stresses in the cell wall could therefore be directly transmitted to MTs, possibly destabilizing MTs that are not aligned along the new orientation of anisotropic mechanical stress and/or stabilizing those that are. However, the observation that the MTs respond more to mechanical stress changes in the absence of CSI1/POM2 than in their presence indicates that MTs have more freedom to align parallel to anisotropic stress patterns when their connection to wall cellulose is diminished. MTs are also known to experience pushing forces due to the polymerization of the CMF, and proteins such as CSI1 and CELLULOSE SYNTHASE-MICROTUBULE UNCOUPLING act to stabilize MTs influencing their organization^38^. Absence of CSI1 might result in MTs being exposed to reduced basal stresses, allowing them to respond better to mechanical stresses. This supports the hypothesis that MTs by themselves could act as a component of a mechanical stress sensor^13^ and that MTs are not caused to align due to their attachment to CMFs via the CSC. It should be noted that the larger cell size and simpler cellular morphologies in *pom2-8* could result in inherently more mechanical stresses present in these cells, priming the cells to respond better to changes in mechanical stresses^31^. MTs are not detached from the PM in the *pom2-8* mutant and are potentially still linked to the cell wall by other proteins.

Our hypothesis that the association of CSC to MTs hinders MT response to mechanical stress is supported by the observation that transient changes to mechanical forces by ablation and compression stop CSC movement abruptly, followed by a reduction in the density of the CSC during which changes in MT orientation are also observed. This is consistent with our previous observation of hyperaligned MTs in cells treated with isoxaben that depletes plasma membrane-localized CSC^4^. At later time points, CSCs migrate along the MTs despite being sparsely distributed. On the basis of these results, we show the existence of a feedback loop in which transient increase in anisotropic mechanical stress causes depletion of CSC from the plasma membrane that aids MT rearrangements along the new direction of mechanical stress, facilitating CMF deposition in similar orientations, thereby alleviating the new mechanical stress. Our results are mainly focused on understanding immediate aspects of mechanical stress transduction and the subsequent MT response. The transient decrease in CSC density upon mechanical perturbation suggests that initial mechanical reinforcement of the cell wall could be mediated by other cell wall components in addition to cellulose, with the existing CMF network acting as a scaffold for mechanical reinforcement. Future experiments on long-term imaging of CSCs and visualization of other cell wall components upon mechanical perturbation will provide further insights into how plants control the tensile and anisotropic properties of their cell walls.

On the basis of our results, we propose that stabilization of MTs along the principal directions of anisotropic mechanical stress could help strengthen established patterns of wall reinforcement, such as during lobe development, while disturbance of this linkage to the wall could provide the noise allowing for greater adaptive response to new inputs, both mechanical and developmental^12^—a principle similarly applicable to mammalian systems^39^.

## Methods

### Plant material, growth conditions and treatments

*Arabidopsis* (Col-0) expressing p35S::Lti6B-GFP^40^ or pCESA3::GFP-CESA3^41^ were crossed with p35S::mCHERRY-TUA5^42^. Both lines were crossed with *csi1-1/pom2-8* (SALK_136239). The dual-labelled PM-MT line was crossed with *prc1-1*^30^. F3 generations of crossed lines were used throughout this study. Seeds were surface-sterilized using 1.25% sodium hypo-chloride (Carl Roth) supplemented with 0.1% Triton X-100 (Millipore Sigma) for 10 min, followed by thorough washing with sterile water. Sterile seeds were stratified for 3 d at 4 °C in the dark. Plants were grown in a climate cabinet running at a 16/8 h long-day cycle (100 µE) with 21/19 °C day/night temperatures. Long-term cell tracking experiments demanded single embryonic cotyledons to be excised from seeds^43^, placed in custom-made imaging chambers, covered by a layer of 0.5% micro-agar and grown in the climate cabinet. The custom-built imaging chambers were mounted on glass coverslips (grade 1.5H, 170 µm thickness, Carl Roth). To enable cell tracking over 96 h, the same region of cells was imaged and subsequently stitched. PCs located proximal to the petiole and half-way between the midrib cells and the cotyledon periphery were recorded every 24 h similarly to Eng et al^8^. In-between time points, imaging chambers were placed in the climate cabinet. Half-strength Murashige and Skoog (½MS) plates were made from MS salt (Millipore Sigma), adjusted to pH 5.7 using KOH and solidified with 0.8% micro-agar (Duchefa Biochemie). On the day of the experiment, cotyledons were dissected from the seedlings and placed under a layer of 1% agarose with their adaxial side facing the coverslip. For short-term oryzalin treatments, dissected cotyledons were incubated for 4 h in 0.2% DMSO (mock) or 20 µM oryzalin.

### Ablation and compression experiments

Experiments were performed using dissected cotyledons expressing Lti6B-GFP and mCHERRY-TUA5 (96 h old) or GFP-CESA3 and mCHERRY-TUA5 (48 h old). Ablations were performed under a stereoscope by incising the cotyledon edge with the sharp tip of a syringe needle. *Z*-stacks of PCs surrounding the ablation site were recorded for 6 h with 2 h intervals. For compression experiments, the cotyledons were placed between a glass cover slide (26 × 76 mm, Marienfeld Superior) and another glass coverslip (24 × 60 mm, Carl Roth) made adherent by adding a thin layer of high-vacuum silicone grease (Dow Corning, Millipore Sigma) along the long sides of the coverslip. This procedure formed a channel with the cotyledons in the middle. Before compression, the channel was filled with water and air bubbles were removed to provide a stable, moist environment. Compression was carefully applied using a toothpick until the cotyledons were visibly pressed against the coverslip. After leaking water was removed with filter paper, the channel was sealed along the short sides of the coverslip by adding more silicone grease. For releasing the compression, we carefully lifted the glass coverslip at their short sides with a syringe needle until the cotyledons were not pressed against the coverslip anymore. Intruding air bubbles were subsequently removed, and the channel was sealed again using silicone grease. For the dual-labelled PM-MT marker lines, a laser-scanning confocal microscope was used, whereas a spinning disk confocal microscope was used for the dual-labelled CSC-MT marker lines. Samples were placed in the climate cabinet between imaging sessions.

### Microscopy and imaging

The spinning disk microscope consisting of a CSU-X1 spinning disk head (Yokogawa) attached to an Eclipse TI inverted microscope (Nikon) was further equipped with the Nikon Perfect Focus system and a Prime 95B sCMOS camera (Photometrics). For imaging PM markers, a Plan Apo ×60 water-immersion objective (Numerical Aperture (NA), 1.2) was used, while a CFI Apo TIRF ×100 oil-immersion objective (NA, 1.49; Nikon) was used for imaging GFP-CESA3. GFP-labelled markers were excited using a 491 nm solid-state laser (Gataca Systems; approximately 10 mW output) and detected using a 525 nm emission filter with a 50 nm band pass (525/50, Chroma). Fluorescence of mCHERRY-TUA5 was excited using a 561 nm solid-state laser (Gataca Systems; approximately 10 mW output) and detected using a 630/75 emission filter (Chroma). Data acquisition was performed using Metamorph software (Molecular Devices, Meta Imaging Series 7.7). Typical image exposure times were 300–500 ms for mCHERRY-TUA5, 500–800 ms for GFP-CESA3 and 500 ms for Lti6B-GFP. Time-lapse images were recorded for a duration of 16 min with 1 min intervals, and between 5 and 20 *z*-planes were collected with a step size (Δ*z*) of 0.3 µm.

The scanning confocal was a Leica SP5 imaging system. GFP was excited with a 488 nm Argon laser, and the emission was collected at 498–550 nm. Fluorescence of mCherry-TUA5 was excited using a 561 nm diode laser, and the emission was detected at 571–615 nm. Imaging was performed using an HC PL APO ×63 water-immersion objective (NA, 1.4). Data acquisition was performed using the LAS AF software.

### Image processing

All recorded *z*-stacks were surface-projected using a custom-made MATLAB code (https://github.com/DrReneSchneider/Smooth-Manifold-Projection-Tool.git). Briefly, the code extracts the *z*-position of the maximal intensity of a reference ‘surface’ marker (here Lti6B-GFP or mCHERRY-TUA5) for each *x*-*y*-pixel of the image stack. Both markers localize predominantly to the PM or the cell cortex, respectively, and thus represent reliable reference markers to extract the ‘true’ (cortical) cell surface. Applying the in-built MATLAB functions ‘smooth.m’ and ‘envelope.m’ to the (three-dimensional) distribution of *z*-positions of maximal intensities enabled the reconstruction of a smoothened, two-dimensional manifold corresponding to the cell surface. This manifold was used to first extract the intensity of the reference marker directly at the cell surface and secondly to apply the same extraction map on a stack containing the target marker (here GFP-CESA3). This approach, yielded the ‘true’ PM and cortical MT signals, respectively, coupled to the ‘true’ GFP-CESA3 signals (originating from the same position in the recorded stack), largely suppressed deep-tissue signals, that is, the bright and mobile Golgi stacks and cytoplasmic mCHERRY-TUA5 fluorescence. The code additionally includes the possibility of performing surface-based maximum-intensity projections. Here, a maximum-intensity projection is performed that starts at the determined cell surface progressing a short distance (typically 3 pixels or 13 approximately 1 µm) into the cell interior. Such projections yielded superior MT and particularly improved CESA3 information and signal-to-noise at the cell surface as compared with the widely used whole-stack maximum-intensity projections. Projected images were further processed to reduce background using the in-built function ‘subtract background’ (rolling ball radius between 5 and 15 pixels) of the Fiji software^44^. In the projected time series, minor drift corrections were performed using the ‘MultiStackReg’ plugin^45^.

To extract individual foci of CESA3, we employed the ‘ThunderSTORM’ plugin^46^. Briefly, the image stacks were filtered using a wavelet filter (B-spline, order 3, scale 2.0), the locations of the foci were approximated using the 8-neighbourhood local maximum method, and the subpixel locations of the foci were determined using the ‘Integrated Gaussian’ point-spread-function with a fitting radius of 3 pixels and an initial sigma of 1.6 pixels. The results were visualized with no additional magnification (×1.0) using the ‘average shifted histogram’ method. To refine the quality of the extracted foci, only foci with a reasonable parameter set were considered, that is, a non-zero offset, a localization error (uncertainty) between 5 and 60 nm, a sigma of 50–500 nm and an intensity below 500,000 (integrated value). In the resulting image stack, the selected foci were replaced by a Gaussian, with its sigma equalling the localization error. Finally, the stack was time-averaged to condense it into a single-slice image.

### Image analysis

To measure the speed of CESA3 foci migrating in the PM, the surface-projected and spot-detected stacks were analysed with the open-source software FIESTA^47^, using the in-built high-throughput, randomized-kymograph method ‘kymograph evaluation’. In the experiments that examined the presence and behaviour of CESAs in the plasma membrane of cotyledon cells over several days, careful attention was paid to the nature of movement of the CESA foci, that is, SMACCs were filtered out by intensity and velocity. In contrast, the experiments involving mechanical manipulation (compression and ablation), velocity and continuity of movement were investigated with the FIESTA software and used as the key discriminator (see Fig. 5f). To quantify the density of CESA3 foci, the output of the ThunderSTORM spot detection, which comprised a list of all detected foci in each slice of the image stack, was used. The total number of CESA3 foci present in each cell throughout the 15 min imaging period was normalized to the projected areal size of the cell (measured manually using the region tools of Fiji) and the number of slices in the image stack.

To accurately assess the MT-CSC co-localization, threshold-based methods were found not to be reliable due to the large fluctuations in fluorescence of the MTs and the CESA3 foci across individual cells but also across the entire field of view. Instead, object-based co-localization was used, which required the extraction of MT features. The ThunderSTORM spot-detection algorithm was therefore also applied to the MT recordings, yielding high-quality images (Supplementary Fig. 1g–i) that were compared to CESA3 images using the co-localization plugin ‘JaCoP’^48^, outputting Pearson’s and Mander’s coefficients. Pearson’s coefficients were tested for significance (*P* < 0.001) using the Costes’ randomization option in JaCoP.

### Contour analysis

A MATLAB script to quantify the correlation of MT and CESA3 signals with the local curvature of the cell contour was published previously^8^ and can be downloaded from GitHub (https://github.com/DrReneSchneider/Matlab-Contour-Analysis). Briefly, projected and time-averaged images of PMs and MTs or MTs and CESA3s were compared. In the first case, PM images were used to extract cell contours using MATLAB’s in-built ‘watershed.m’ function, whereas in the second case, cell contours were generated manually using Fiji’s region tools. Care was taken to accurately determine the cell boundaries in the respective MT images. For this purpose, unprocessed MT *z*-stacks were focused into deeper *z*-slices where the cell outlines (cross-sections) were well resolved. Subsequently, the MATLAB script was applied, creating a margin starting at the cell contour progressing inwards by 1.8 µm for dual CESA3-MT recordings and by 1.2 µm for PM-MT recordings. MT and CESA3 intensities were measured only in the margin, and for each point along the cell contour, only the local MT intensity was measured. Local curvature, defined as the reciprocal of the radius of a circle, was measured by fitting the local cell contour (a stretch of 1.8 µm and 1.2 µm along the contour for CESA3-MT and PM-MT images, respectively) to a circle using the ‘circfit.m’ function^49^ (from Izhak Bucher, MathWorks File Exchange, 1991). Negative (concave) curvatures (indenting regions of the PC) were assigned if the centre of the fitted circle was outside of the cell contour, and positive (convex) curvature (protruding regions) otherwise. On the basis of all points of the cell contour, the MT-/CESA3-curvature correlation coefficient was determined. Negative correlation coefficients reflect MTs/CESA3s localizing more to indenting domains, while positive correlation coefficients indicate the inverse (MTs/CESA3s localizing more to protruding domains). Cell borders along walls shared with stomata were ignored. The local orientation and anisotropy of MTs and CESA3 trajectories were analysed in the local vicinity of each point of the cell contour by incorporating the published code of FibrilTool^32^ into our MATLAB code.

### Statistical analysis and data display

Quantified parameters were plotted and analysed using the Plots-Of-Data, PlotTwist and SuperPlotsOfData web apps^49,50^. Unless stated otherwise, Welch’s unpaired, two-sided *t*-test was performed using the GraphPad website (https://www.graphpad.com/quickcalcs/ttest1.cfm). Unless stated otherwise, plots display means ± 95% confidence intervals. See the source data file for detailed information on sample and measurement numbers.

### Statistics and reproducibility

Detailed numbers are documented in the Source Data files. Briefly, for the plots shown in the main text: Fig. 1d–f, in brackets: number of CESA3s (d) and cells (e,f) analysed from 2, 7, 4, 3 and 3 seedlings; Fig. 1g, in brackets: number of CESA3s from 5 seedlings each; Fig. 1i, in brackets: number of cells analysed from 2, 7, 4, 3 and 3 seedlings; Fig. 2c,d, in brackets: number of cells from 6 seedlings; Fig. 2g, in brackets: number of cells from 4 seedlings each; Fig. 2h,i, in brackets: number of cells from 4 seedlings each; Fig. 3e,i–k, in brackets: number of cells from 7, 3 and 4 seedlings for wild type, *pom2-8* and *prc1-1*, respectively; Fig. 4b, in brackets: number of cells from 3, 7 and 6 seedlings for wild type, *pom2-8* and *prc1-1*, respectively; Fig. 4c,e, same cells as indicated in Fig. 4b. Figure 5b,c, in brackets: number of cells from 5 seedlings; Fig. 5f,g, in brackets: number of CSCs from 4 seedlings; Supplementary Fig. 4c, in brackets: number of CSCs from 2, 6, 4, 3 and 3 seedlings; Supplementary Fig. 7b–d, in brackets: number of cells from 5 seedlings. Supplementary Fig. 7e, same cells as indicated in Fig. 5b,c. Supplementary Fig. 7f: same cells as indicated in Supplementary Fig. 7b,c. Supplementary Fig. 8d, in brackets: number of cells from 4 seedlings.

### Reporting summary

Further information on research design is available in the Nature Research Reporting Summary linked to this article.

## Supplementary information

Supplementary Information Supplementary Figs. 1–8.

Reporting Summary

Supplementary Video 1 Detection of plasma membrane-localized cellulose synthase complexes.

Supplementary Video 2 Microtubule-independent cellulose synthase complex migration immediately post dissection.

Supplementary Video 3 Microtubule depolymerization results in perturbation of cell shape-dependent cellulose synthase patterning.

Supplementary Video 4 Genetic uncoupling of cell shape-dependent cellulose synthase patterning.

Supplementary Video 5 Cellulose synthase complex migration abruptly stops immediately after ablation.

Supplementary Video 6 Changes in cellulose synthase dynamics and patterning post ablation.

## Data Availability

The imaging datasets that serve as the basis for the figures in this paper can be obtained on Zenodo (https://zenodo.org/record/6660991; 10.5281/zenodo.6660991). All additional image datasets are available upon request from R.S. and A.S. Source data are provided with this paper.

## Source data

Source Data Fig. 1 Statistical source and data.
Source Data Fig. 2 Statistical source and data.
Source Data Fig. 3 Statistical source and data.
Source Data Fig. 4 Statistical source and data.
Source Data Fig. 5 Statistical source and data.
Source Data Supplementary Fig. 4 Statistical source and data.
Source Data Supplementary Fig. 7 Statistical source and data.
Source Data Supplementary Fig. 8 Statistical source and data.

## Code availability

Computer code for generating surface-based maximum-intensity projections can be accessed from GitHub (https://github.com/DrReneSchneider/Smooth-Manifold-Projection-Tool.git). Further assistance with coding is recommended and can be provided by R.S. and A.S upon request.
