# Supplementary material for: Tethering of cellulose synthase to microtubules dampens mechano-induced cytoskeletal organization in Arabidopsis pavement cells
Source: Nat Plants. 2022 Aug 18;8(9):1064–73. doi: 10.1038/s41477-022-01218-7 (PMC9477734; doi:10.1038/s41477-022-01218-7)
Supplement: Supplementary file 1 — Supplementary Figs. 1–8. [file 41477_2022_1218_MOESM1_ESM.pdf]

nature portfolio

https://doi.org/10.1038/s41477-022-01218-7

Supplementary information

Tethering of cellulose synthase to microtubules dampens mechano-induced cytoskeletal organization in *Arabidopsis* pavement cells

In the format provided by the authors and unedited

SUPPLEMENTARY FIGURES

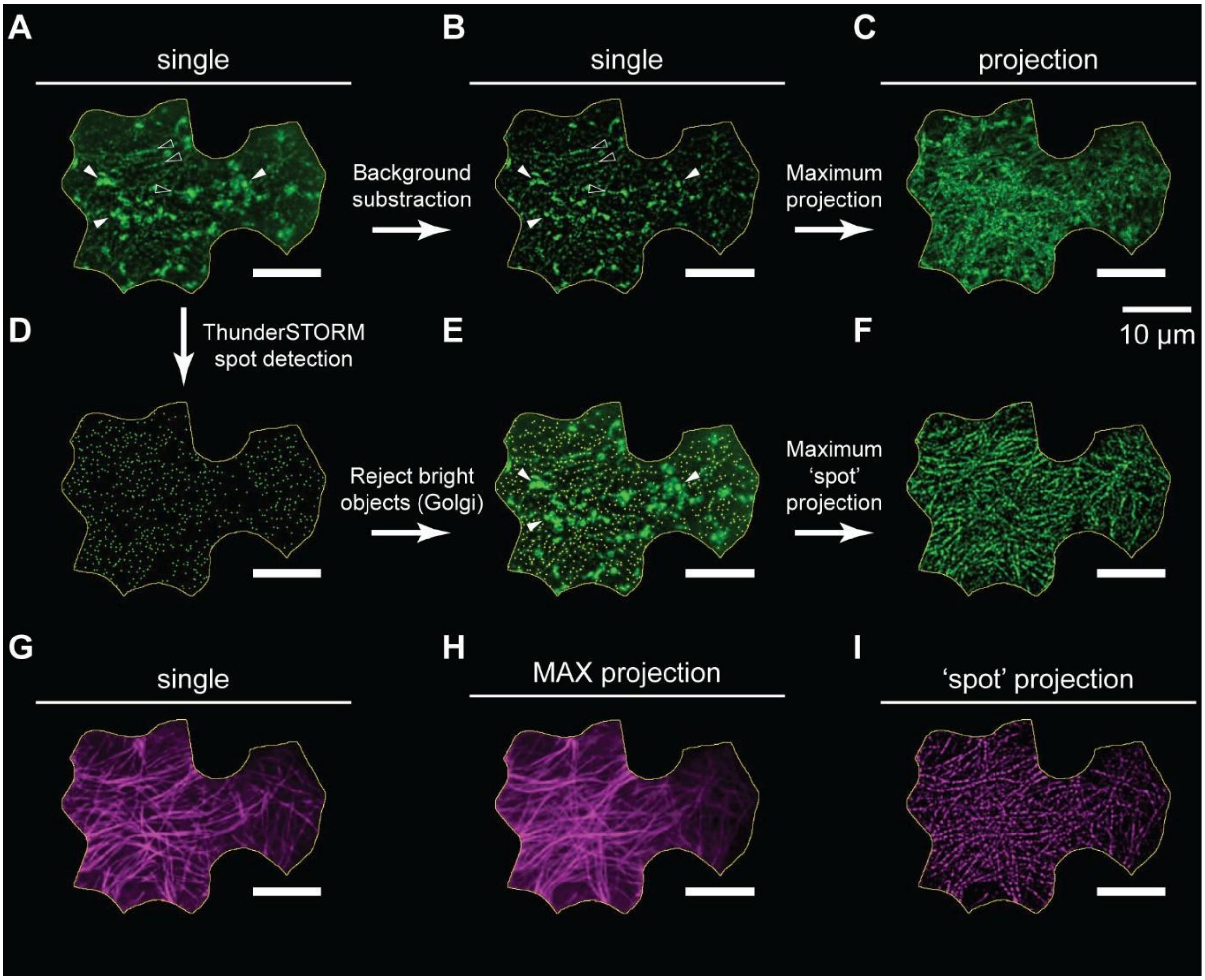

Supplementary Figure 1 | Post-processing workflow for confocal *z*-stack recordings of dual-labelled cellulose synthases and microtubule marker lines. (**A**) Single snapshot of an example cell at the 48-hour time point showing CSC tracks (empty arrowheads) and bright and mobile Golgi bodies (filled arrowhead). (**B**) Background subtraction partly suppresses the bright Golgi signal (filled arrowheads) while maintaining the CSC tracks (empty arrowheads). (**C**) Maximum projection of the background-subtracted single snapshots yields the bright Golgi movement pattern to overlap almost entirely with the finer CSC tracks. (**D**) Using the spot-detection algorithm of the 'ThunderSTORM' Fiji plugin allows fluorescent foci to be approximated by two-dimensional Gaussians and replaced by smaller foci. (**E**) Ignoring the Golgi (using intensity thresholding; filled arrowheads) allows the visibility of the CSC foci to be strongly enhanced. (**F**) Maximum projecting the detected spots yields fine CSC trajectories. (**G-I**) The algorithm was applied also to MTs. Single MT snapshots (G) and maximum projections (H) yield sufficient contrast for analysis. Projecting the spot-detected snap shots (I) yields supreme image analysis data that can be used for object-based analyses (e.g., co-localization). Bars = 10 µm.

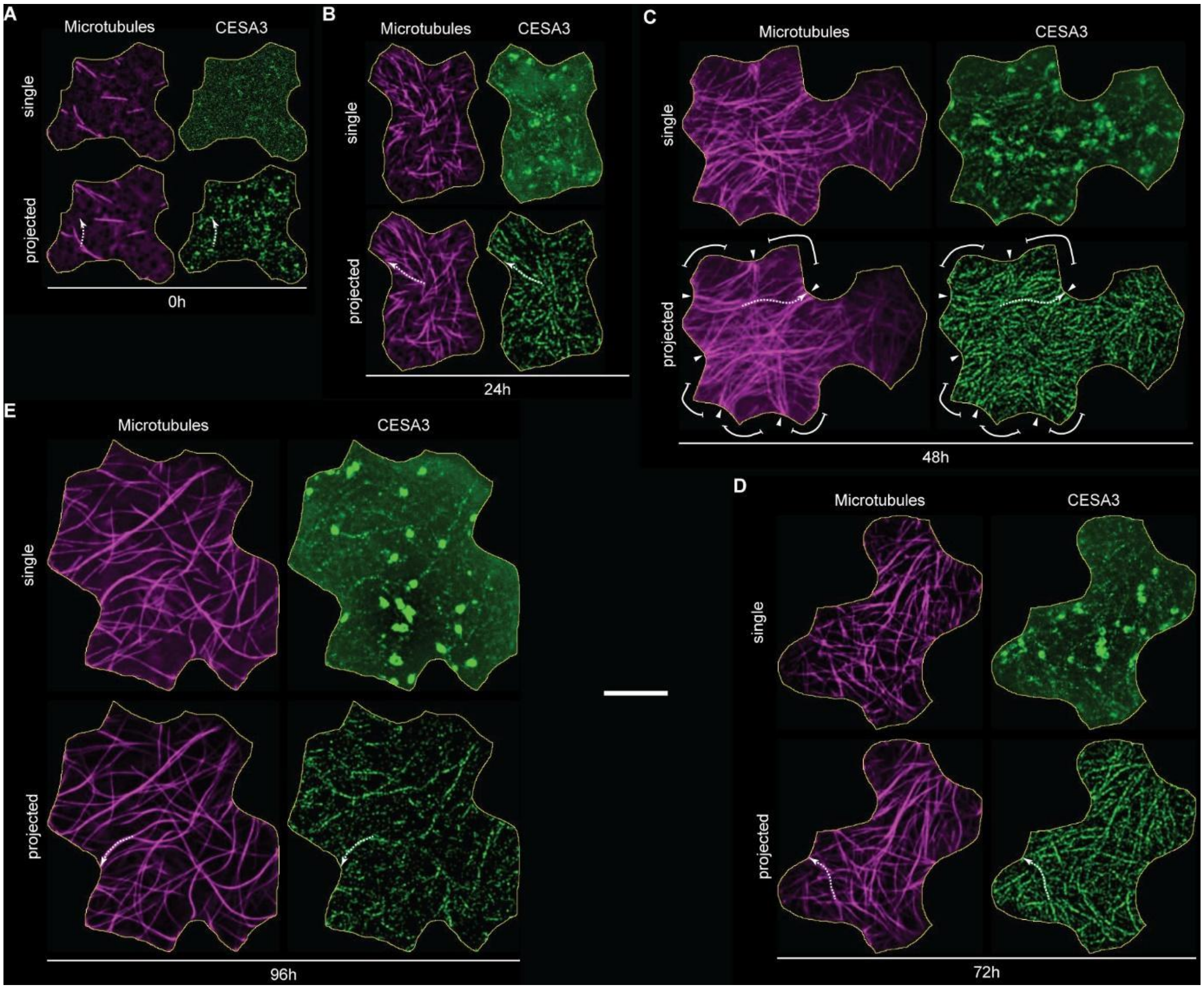

Supplementary Figure 2 | Split-colour projections of microtubules and cellulose synthases in single cotyledon pavement cells at 0, 24, 48, 72, and 96 hours after dissection. (**A-E**) Single snapshots (top) and projected images (bottom) for MTs (magenta, left) and CESA3 (green, right) at 0-hpd (A), 24-hpd (B), 48-hpd (C), 72-hpd (D), and 96-hpd (E) after seed dissection. Bar = 10 µm. Dotted arrows depict regions along which kymographs were taken (see Fig. 1B in the main text). At 48 hours, MTs strongly correlate with negatively curved regions (indentations) as indicated by the filled arrowheads. Regions with positive curvature (protrusions), however, are less populated with MTs. This pattern is slowly lost over the following time points. Similar data has been collected for 2, 7, 4, 3, and 3 seedlings for 0-, 24-, 48-, 72-, and 96-hpd. Bar = 10µm.

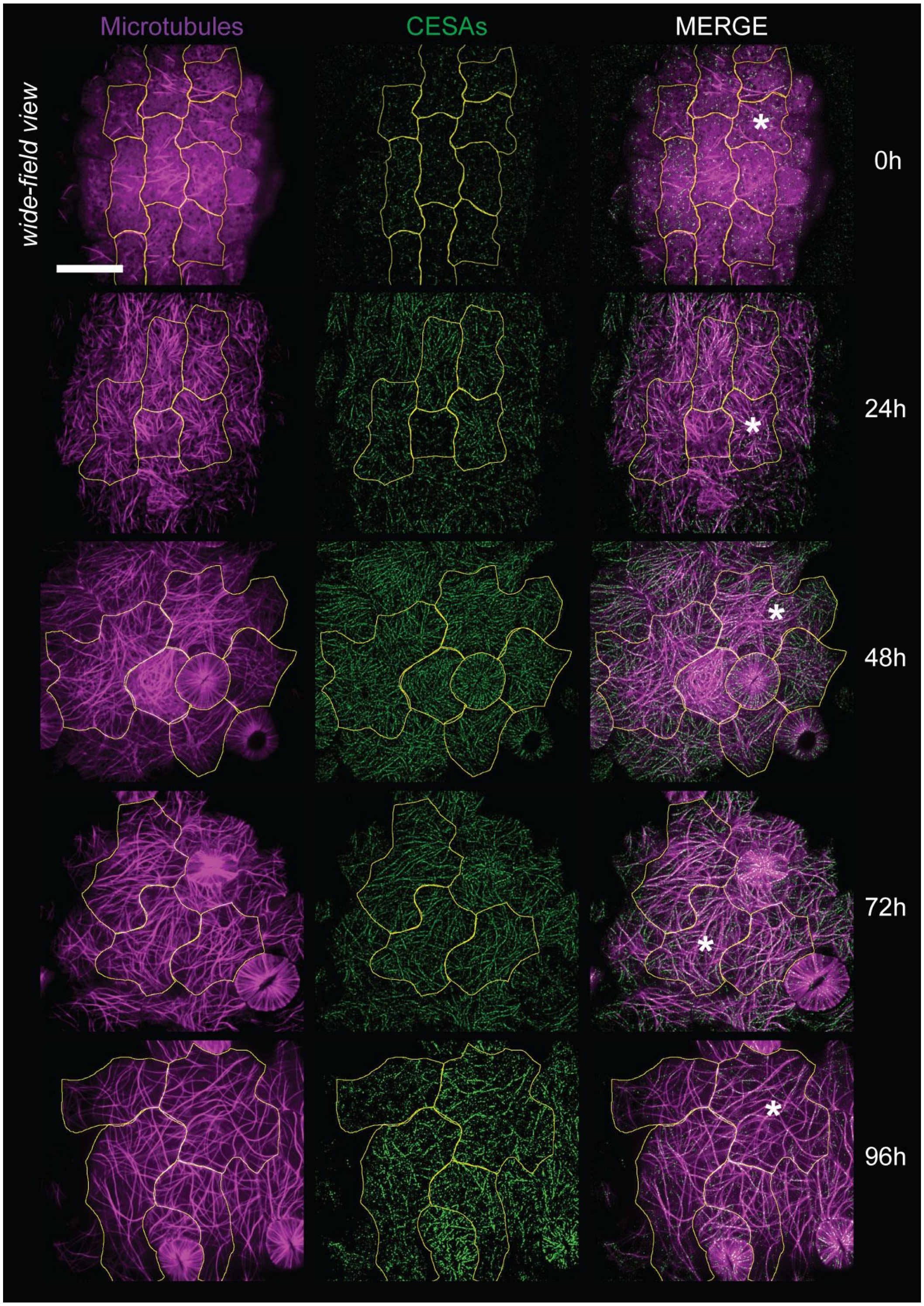

Supplementary Figure 3 | Field of views of split-colour projections of microtubules and cellulose synthases in single cotyledon pavement cells at 0, 24, 48, 72, and 96 hours after dissection. Time-averages of surface-projected microtubules (magenta, left), spot-detected CESA3 foci (green, middle), and merged images (right) showing the outlines of wild-type cells selected for analysis (in yellow). Asterisks mark the cells shown in Fig. 1. All cells for which the entire cell border and periclinal surface were visible were included in the analysis. Similar data has been collected for 2, 7, 4, 3, and 3 seedlings for 0-, 24-, 48-, 72-, and 96-hpd. Bar = 20µm.

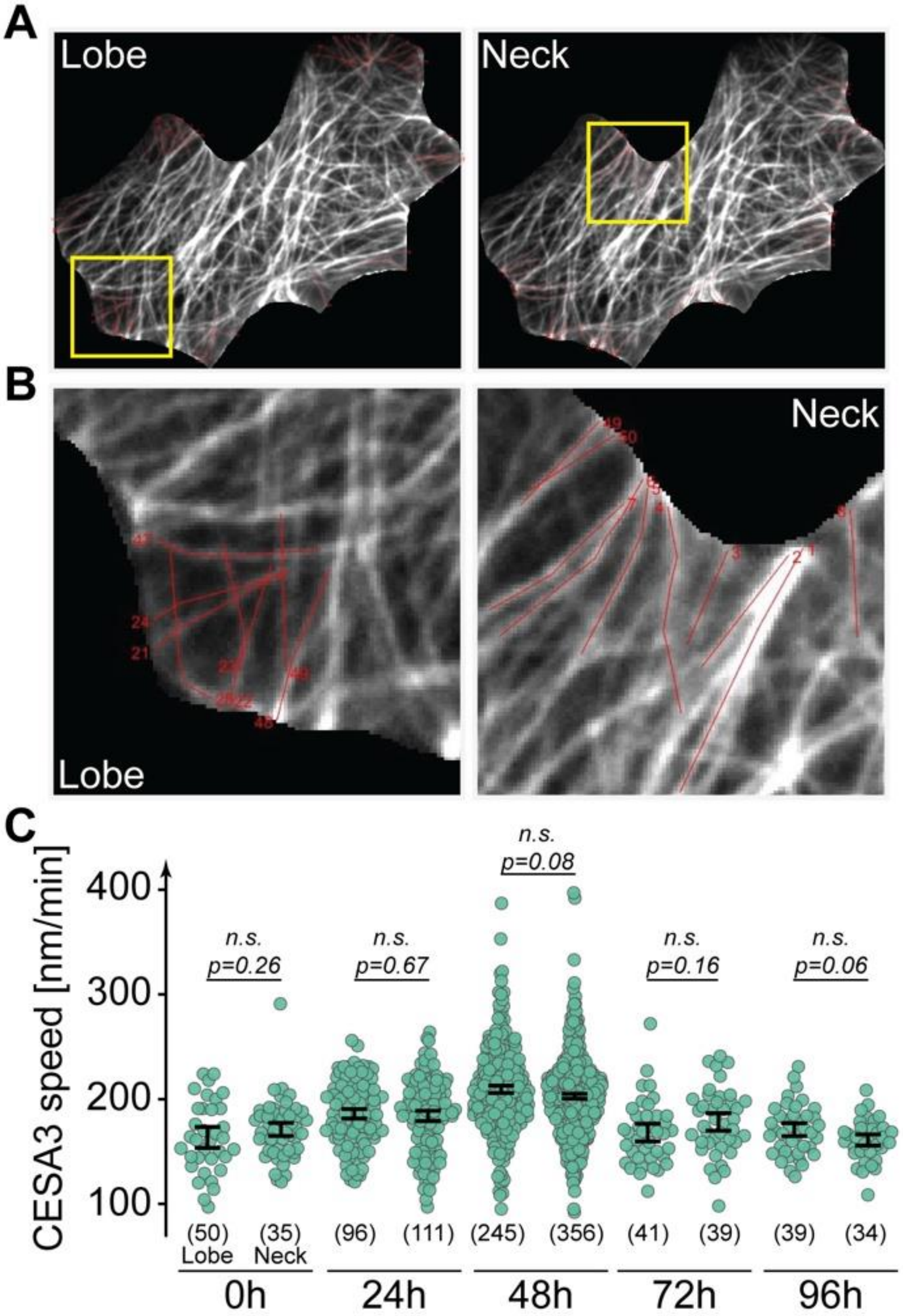

Supplementary Figure 4 | Quantification of CESA3 speeds in lobes and necks during cotyledon development from 0hpd to 96hpd. (**A**) Screenshot of an example cell at 48hpd showing lobe- (left) and neck-localized (right) MTs selected for kymograph analysis of their associated CESA3 signals. (**B**) Zoomed-in region of yellow boxes in (A). (**C**) CESA3 speeds in cotyledon cells at 0hpd, 24hpd, 48hpd (data from main text Fig. 1D), 72hpd, and 96hpd were measured using the FIESTA software (44). In brackets: number of CESA3s measured from 14, 50, 22, 11, and 12 cells. Means ± 95% confidence intervals. Welch's unpaired *t*-tests (two-tailed, *p*-values) between lobes and necks for each time point. See statistics and reproducibility in the methods section.

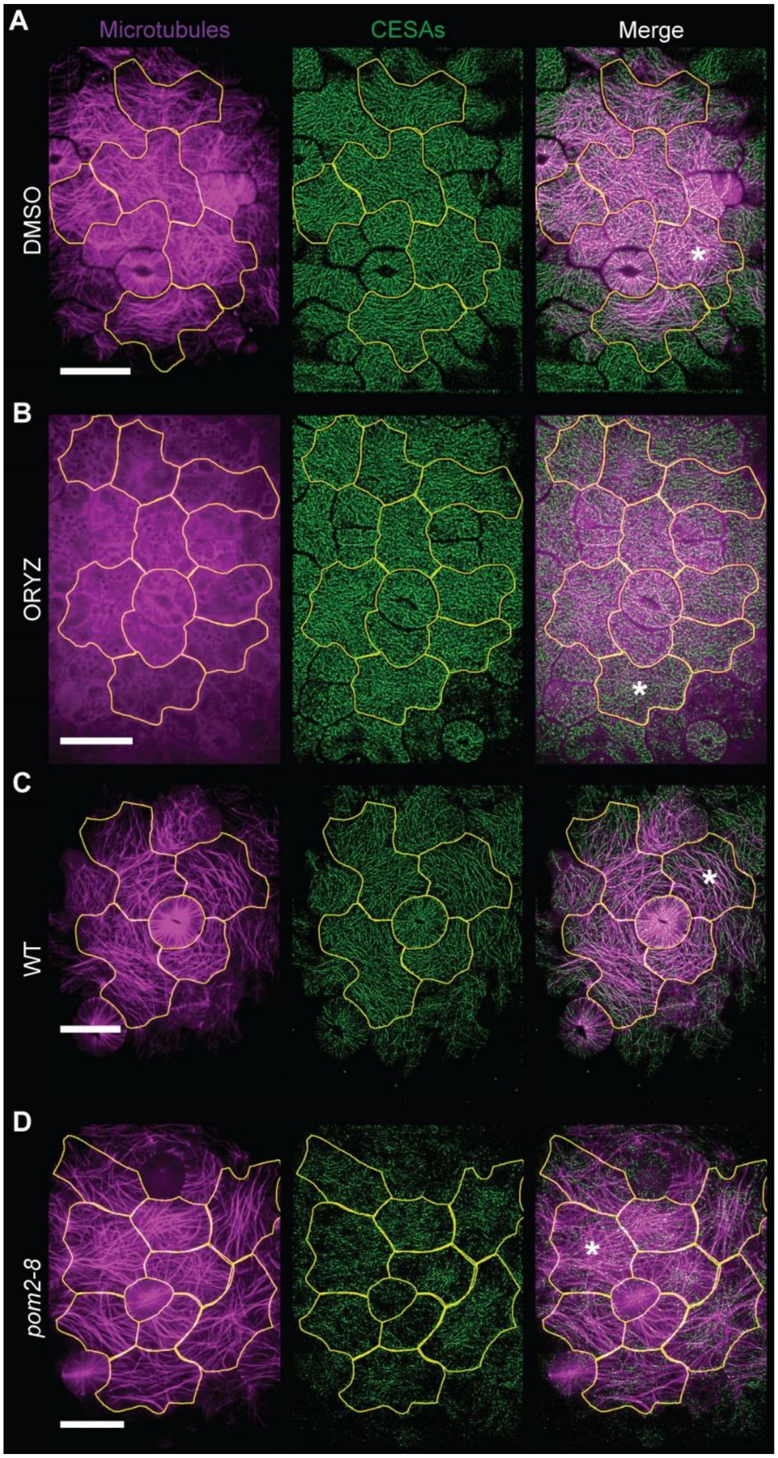

Supplementary Figure 5 | Field of views of split-colour projections of microtubules and cellulose synthases in single cotyledon pavement cells at 48 hours after dissection. (**A-D**) Wide-field images supporting data shown in Figure 2 (main text) for mock-treated (A) and Oryzalin-treated wild type (B), and for wild-type (C) and *pom2-8* mutant (D) cells. Time-averages of surface-projected microtubules (magenta, left), spot-detected CESA3 foci (green, middle), and merged images (right) showing the outlines of the cells selected for analysis (in yellow). The cells shown in Figure 2 of the main text are labeled by an asterisk. Similar data was obtained for 6 (A,B) and 4 (C,D) seedlings. Bars = 20 µm.

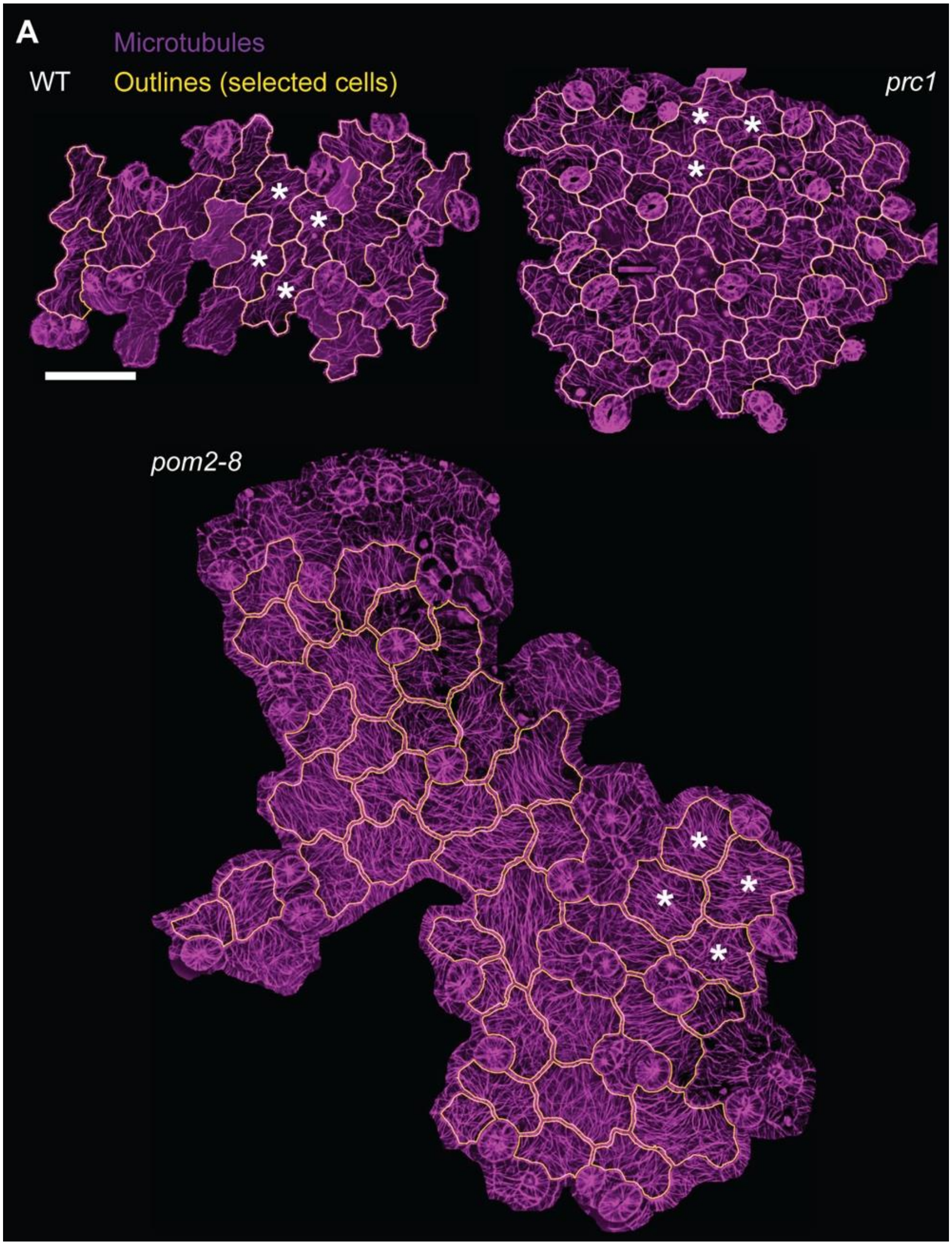

Supplementary Figure 6 | Field of views of surface-projected microtubules and segmented cell outlines at 96hpd for wild-type, *pom2-8,* and *prc1-1* mutant cells. Time-averages of surface-projected microtubules (magenta) and segmented cell outlines (yellow) of the cells selected for analysis. The cells shown in Figure 3 of the main text are labeled by an asterisk. All cells for which the entire cell border and periclinal surface were visible were included in the analysis. Note that the only cells being rejected from our analyzes were either i) dividing, ii) stomata, iii) cells that were not captured for the entire 96-hour time course (i.e., cells at the margin of the imaged region) and iv) cells that grew significantly into the z-direction causing the surface-projection method to reach its limitation. Similar data was obtained for 7, 3, and 4 seedlings for wild type, *pom2-8*, and *prc1-1*, respectively. Bar = 50 µm.

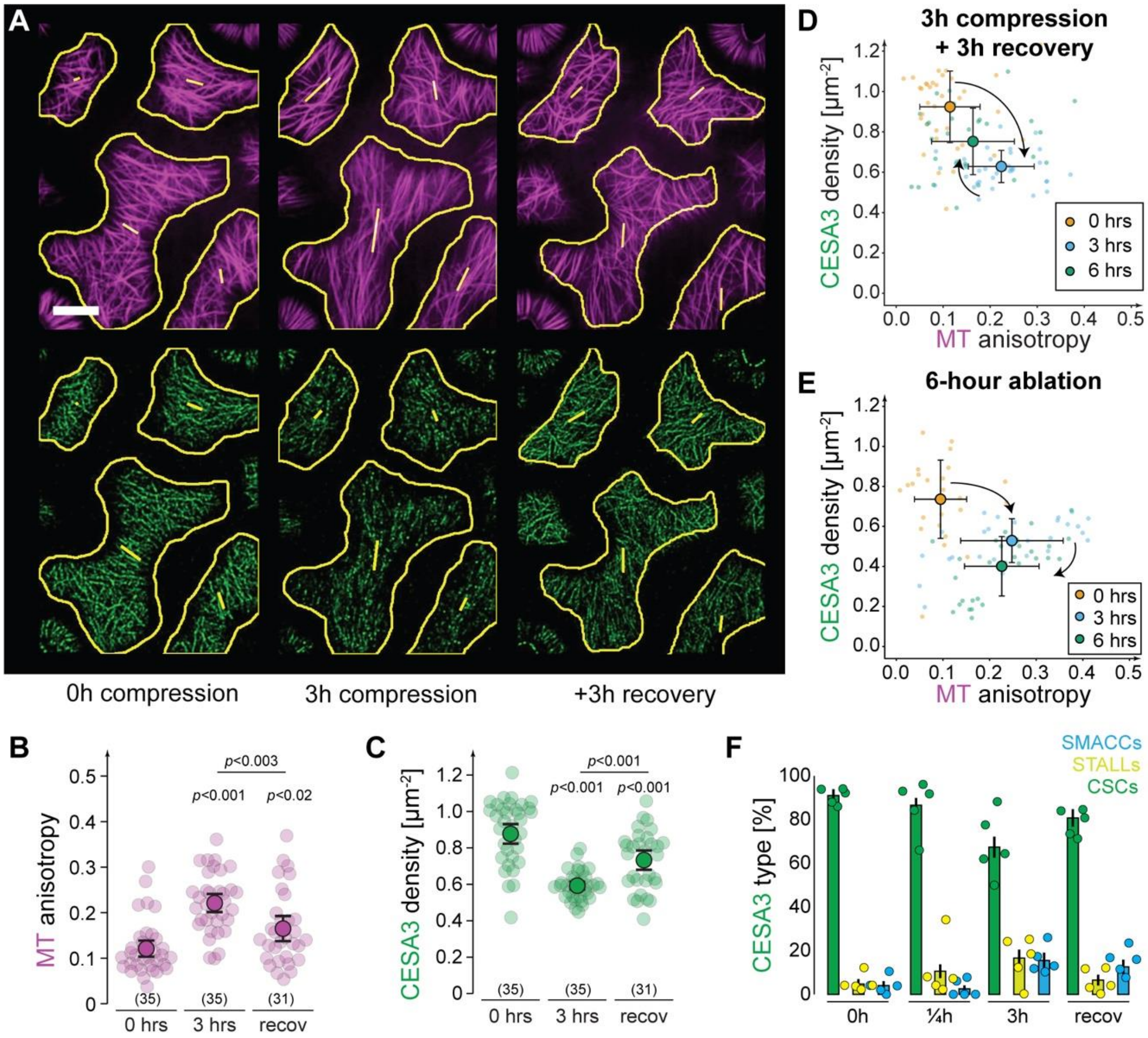

Supplementary Figure 7 | Effects of perturbing cotyledons by compression are reversible. (**A**) Projection of MTs (magenta) and CESA3 (green) in cotyledon PCs at the 96-hour time point at 0 and 3 hours after compression, and after 3 hours of recovery (i.e., 6 hours after start of compression). Cell outlines added manually (yellow). MT orientation and anisotropy indicated by the yellow line inside the cells. Bar = 10 µm. (**B-C**) MT anisotropy (B) and CESA3 density (C) in compressed cells at 0 and 3 hours post compression, and after recovery. Means ± 95 % confidence intervals. Welch's unpaired *t*-test (two-tailed, p-values) relative to 0-hour and between time points. (**D**) Scatter plot of 3-hour compression + 3-hour recovery data (B and C combined). Small dots represent individual cell values, big dots represent the ensemble means ± standard deviations at the 0- (orange), 3- (blue), and 6-hour (green) time point. Upon release of mechanical compression (after 3 hours), MT anisotropy and CESA3 cortical density recovers partially (compare 0-hour time point). (**E**) Scatter plot of 6-hour ablation data (B and C from main text Fig. 6 combined). Small dots represent individual cell values, big dots represent the ensemble means ± standard deviations at the 0- (orange), 3- (blue), and 6-hour (green) time point. (**F**) Fraction of CSCs, STALLs, and SMACCs over the time course of the compression and recovery experiment. Means ± standard error of the mean. See statistics and reproducibility in the methods section.

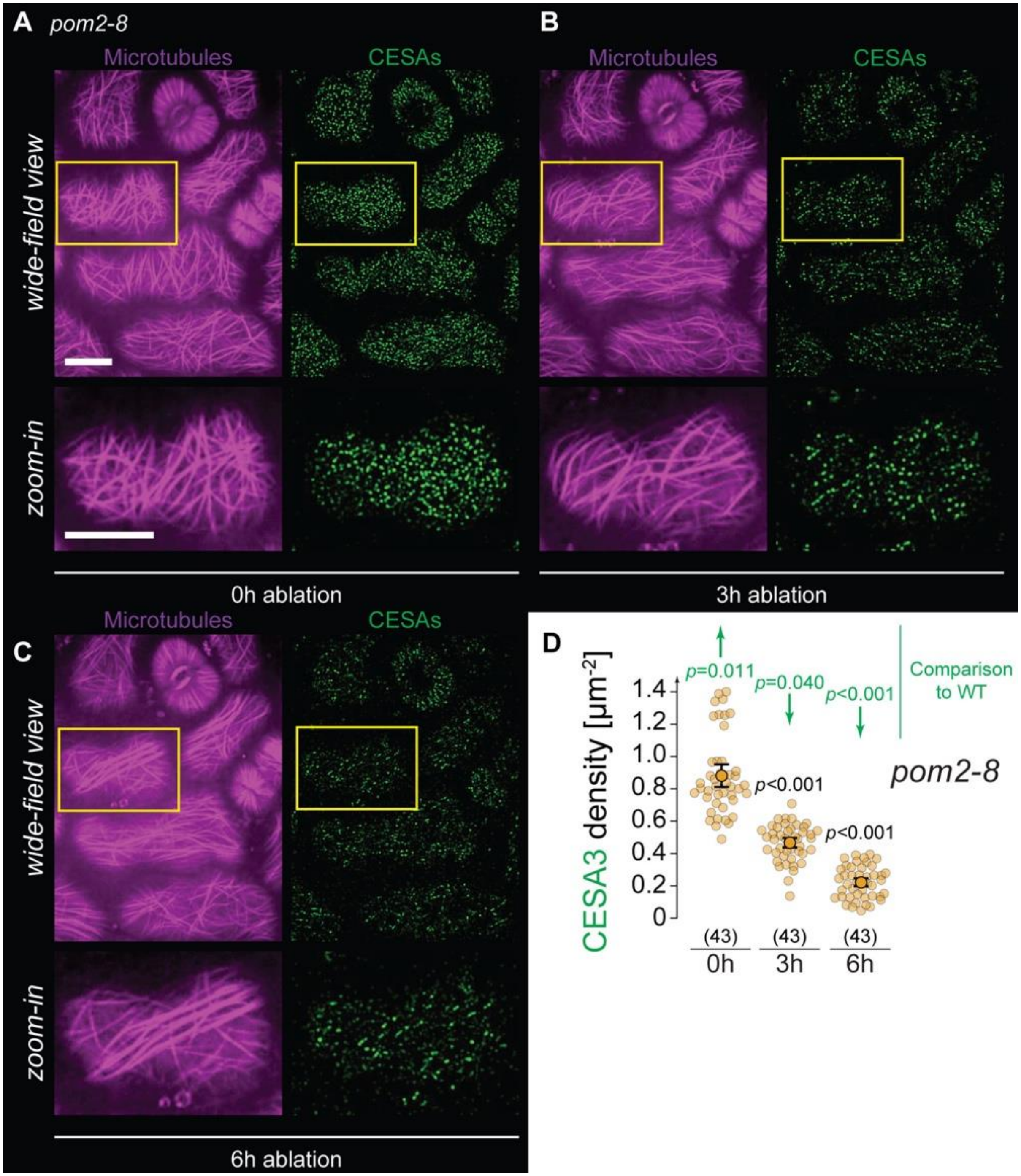

Supplementary Figure 8 | CESA3 density drops significantly more in *pom2-8* compared to wild type after tissue ablation. (**A-C**) Field of views (top row) of surface-projected MTs (magenta, left) and spot-detected CESA3s (green, right) and zoomed-in single-cell images (bottom row; from yellow boxes in upper row) for directly after ablation (0h, A), after 3 hours (B), and after 6 hours (C) in the *pom2-8* mutant background. Bars = 10 µm. (**D**) CESA3 density at the periclinal cell cortex. Similar to wild type, CESA3 density drops from a value slightly higher than wild type at 0h ($p = 0.011$), to values significantly smaller than wild type ($p = 0.04$ and $p < 0.001$) at 3h and 6h after ablation. Means ± 95% confidence intervals. Welch's unpaired *t*-test (two-tailed, *p*-values) for comparisons to wild type for the 0h, 3h, and 6h time points (green, compare main text Fig. 5C) and between time points for *pom2-8* (black). See statistics and reproducibility in the methods section.
