# Supplementary material for: Tethering of cellulose synthase to microtubules dampens mechano-induced cytoskeletal organization in Arabidopsis pavement cells
Source: Nat Plants. 2022 Aug 18;8(9):1064–73. doi: 10.1038/s41477-022-01218-7 (PMC9477734; doi:10.1038/s41477-022-01218-7)
Supplement: Supplementary file 2 — Reporting Summary [file 41477_2022_1218_MOESM2_ESM.pdf]

nature portfolio

Corresponding author(s): Arun Sampathkumar, NPLANTS-220112277A

Last updated by author(s): Jun 19, 2022

Reporting Summary

Nature Portfolio wishes to improve the reproducibility of the work that we publish. This form provides structure for consistency and transparency in reporting. For further information on Nature Portfolio policies, see our Editorial Policies and the Editorial Policy Checklist.

Statistics

For all statistical analyses, confirm that the following items are present in the figure legend, table legend, main text, or Methods section.

n/a	Confirmed	
☐	☒	The exact sample size (n) for each experimental group/condition, given as a discrete number and unit of measurement
☐	☒	A statement on whether measurements were taken from distinct samples or whether the same sample was measured repeatedly
☐	☒	The statistical test(s) used AND whether they are one- or two-sided<br>*Only common tests should be described solely by name; describe more complex techniques in the Methods section.*
☒	☐	A description of all covariates tested
☒	☐	A description of any assumptions or corrections, such as tests of normality and adjustment for multiple comparisons
☐	☒	A full description of the statistical parameters including central tendency (e.g. means) or other basic estimates (e.g. regression coefficient) AND variation (e.g. standard deviation) or associated estimates of uncertainty (e.g. confidence intervals)
☐	☒	For null hypothesis testing, the test statistic (e.g. F, t, r) with confidence intervals, effect sizes, degrees of freedom and P value noted<br>*Give P values as exact values whenever suitable.*
☒	☐	For Bayesian analysis, information on the choice of priors and Markov chain Monte Carlo settings
☒	☐	For hierarchical and complex designs, identification of the appropriate level for tests and full reporting of outcomes
☐	☒	Estimates of effect sizes (e.g. Cohen's d, Pearson's r), indicating how they were calculated

Our web collection on statistics for biologists contains articles on many of the points above.

Software and code

Policy information about availability of computer code

Data collection	We used the commercially available softwares Metamorph Imaging, and Leica LasX to record data from microscopes.
Data analysis	Data was analysed using the following softwares: Fiji/ImageJ (multiple versions), source code of FibrilTool (originally published version from Boudaoud et al., Nature Protocols, 2014) and its batch version, Matlab (R2020b and later versions), and the free tracking software FIESTA (1.05.0000). For contour analyses of microtubule distribution, we used a previously published custom Matlab script (https://github.com/DrReneSchneider/Matlab-Contour-Analysis). We further developed this script to correlate a second fluorescence channel (CESAs) along with the first (microtubules) with cell contour. We also developed a Matlab script that allows surface-based maximum-intensity projections to be generated, which can be downloaded from GitHub (https://github.com/DrReneSchneider/Smooth-Manifold-Projection-Tool). We used the following websites to perform statistical testing and statistical data representation: PlotsOfData (https://huygens.science.uva.nl/PlotsOfData/), PlotTwist (https://huygens.science.uva.nl/PlotTwist/), SuperPlotsOfData (https:// huygens.science.uva.nl/SuperPlotsOfData/), and GraphPad (for t-tests; https://www.graphpad.com/quickcalcs/ttest1.cfm).

For manuscripts utilizing custom algorithms or software that are central to the research but not yet described in published literature, software must be made available to editors and reviewers. We strongly encourage code deposition in a community repository (e.g. GitHub). See the Nature Portfolio guidelines for submitting code & software for further information.

Data

Policy information about availability of data

All manuscripts must include a data availability statement. This statement should provide the following information, where applicable:

- Accession codes, unique identifiers, or web links for publicly available datasets
- A description of any restrictions on data availability
- For clinical datasets or third party data, please ensure that the statement adheres to our policy

The imaging datasets that serve as the basis for the figures in our manuscript were uploaded to a folder on Zenodo (https://zenodo.org/record/6660991; doi: 10.5281/zenodo.6660991). All additional image datasets are available upon request from Dr. René Schneider and Dr. Arun Sampathkumar.

Field-specific reporting

Please select the one below that is the best fit for your research. If you are not sure, read the appropriate sections before making your selection.

☒ Life sciences ☐ Behavioural & social sciences ☐ Ecological, evolutionary & environmental sciences

For a reference copy of the document with all sections, see nature.com/documents/nr-reporting-summary-flat.pdf

Life sciences study design

All studies must disclose on these points even when the disclosure is negative.

Sample size	There was no specific statistical method used to determine sample size, except for the number of CESAs required for accurate velocity determination. This number was determined experimentally by measuring up to 250 CESAs in each of several individual cells. Then, sub-samples were taken with variable sample sizes ranging from 3 up to 250. For each their mean and standard deviation were determined. This procedure resulted in a sample size of about 50 to 100 CESAs above which there was no further significant change in the measured values. Furthermore, we used sample sizes that yielded sufficient statistical power in previous studies from our labs such as in Schneider et al., Plant Cell (2017), Watanabe and Schneider et al., PNAS (2018), Schneider and van't Klooster et al., Nature Communications (2021), and Eng et al., Current Biology (2021).
Data exclusions	No samples or recordings were excluded, except when samples showed (non-linear) drift or other changes during image acquisition that could not be corrected.
Replication	All experiments contain at least three biological replicates (except the 0-hpd time point [CESAs and microtubules in WT] presented in Fig. 1 where only 2 seedlings were available), with multiple cells in each set. All replicates were independent from each other. We performed test experiments prior to image recording and always found full repeatability.
Randomization	Seedlings (and Genotypes) were always randomly distributed during growth and treatment. Kymographs - to measure CESA speeds in FIESTA - were displayed in randomized order with varying scaling to minimize user bias.
Blinding	Blinding was not necessary, because (where possible) we automatized our image analysis (e.g. CESA3 density, Pearson's coefficients, curvature correlations, anisotropy and alignment, lobeyness, cell area, and classification of CESAs into CSCs, STALLs, and SMACCs). If automation was not possible, we used randomized image display (see Randomization above for CESA3 speeds).

Reporting for specific materials, systems and methods

We require information from authors about some types of materials, experimental systems and methods used in many studies. Here, indicate whether each material, system or method listed is relevant to your study. If you are not sure if a list item applies to your research, read the appropriate section before selecting a response.

Materials & experimental systems

n/a	Involved in the study	
☒	☐	Antibodies
☒	☐	Eukaryotic cell lines
☒	☐	Palaeontology and archaeology
☒	☐	Animals and other organisms
☒	☐	Human research participants
☒	☐	Clinical data
☒	☐	Dual use research of concern

Methods

n/a	Involved in the study	
☒	☐	ChIP-seq
☒	☐	Flow cytometry
☒	☐	MRI-based neuroimaging
